# Supplementary material for: Tanzanian goat gut microbiomes adapt to roadside pollutants and environmental stressors
Source: Microbiol Spectr. 2025 Dec 17;14(2):e02036-25. doi: 10.1128/spectrum.02036-25 (PMC12889105; doi:10.1128/spectrum.02036-25)
Supplement: Supplemental material 1 — Tables S1 to S5; Fig. S1 to S6. [file spectrum.02036-25-s0001.pdf]

Supplementary material part 1

Supplementary table 1

Table S1: Number of paired-end reads aligned to databases per sample.

Sample	Total PE reads	PE bacterial reads padMAG+bac+bac_draft	PanRes	ResFinder	ResFinderFG	ResFinderNG	BacMet
Road_1	59376750	33980431	420394	1702	12417	38418	38418
Road_2	54701361	35978722	40077	756	4424	6120	6120
Road_3	74276910	34835928	47242	1895	3214	4405	4405
Road_4	50586987	32533490	33183	739	2599	3647	3647
Road_5	53393436	26353225	25861	1438	2348	2013	2013
Road_6	53000595	25720404	76642	1193	2777	7061	7061
Road_7	83040348	41976111	83800	2761	3399	7965	7965
Road_8	107701916	48492647	86484	2942	5458	8156	8156
Road_9	44478925	20365188	38642	1415	2422	3749	3749
Road_10	44013794	21323386	102250	1476	3666	10414	10414
Road_11	43244773	21909143	76240	1415	2851	7655	7655
Road_12	44246751	21410245	80994	1531	2987	8382	8382
Road_13	42579100	20975707	54970	1386	2290	5300	5300
Road_14	41887319	23020193	8441	1495	2154	850	850
Road_15	43363533	24406081	31937	685	1698	3191	3191
Road_16	44712335	21369272	33040	1230	2199	3066	3066
Road_18	44033436	17741053	9400	1335	2579	527	527
Road_19	43998843	25031761	7490	731	2007	889	889
Road_20	44103430	21526503	85721	972	3217	8199	8199
Rural_26	45624718	23085178	262602	3035	7658	31847	31847
Rural_27	44876093	21643084	3937	840	798	297	297
Rural_28	45940299	24233050	239943	2942	6692	28386	28386
Rural_29	43957574	19658194	3507	723	1373	70	70
Rural_30	44914323	26550123	30682	1237	2141	2973	2973
Rural_31	45108727	17853216	31367	873	2169	3230	3230
Rural_32	44420523	28201695	526441	4799	14490	61649	61649
Rural_33	48876195	27502211	22040	1178	1857	2159	2159
Rural_34	56655886	24750162	13309	1014	2039	1199	1199
Rural_35	47830470	25389656	4986	725	1186	375	375
Rural_36	54739672	24488817	22286	1299	1760	2839	2839
Rural_37	44050083	27423862	618155	3280	16899	67800	67800
Rural_38	45527355	23144043	5204	765	1450	346	346
Rural_39	45844312	24041519	6465	1634	1152	486	486
Rural_40	45348713	23954705	4080	695	1133	279	279

Supplementary figure 1

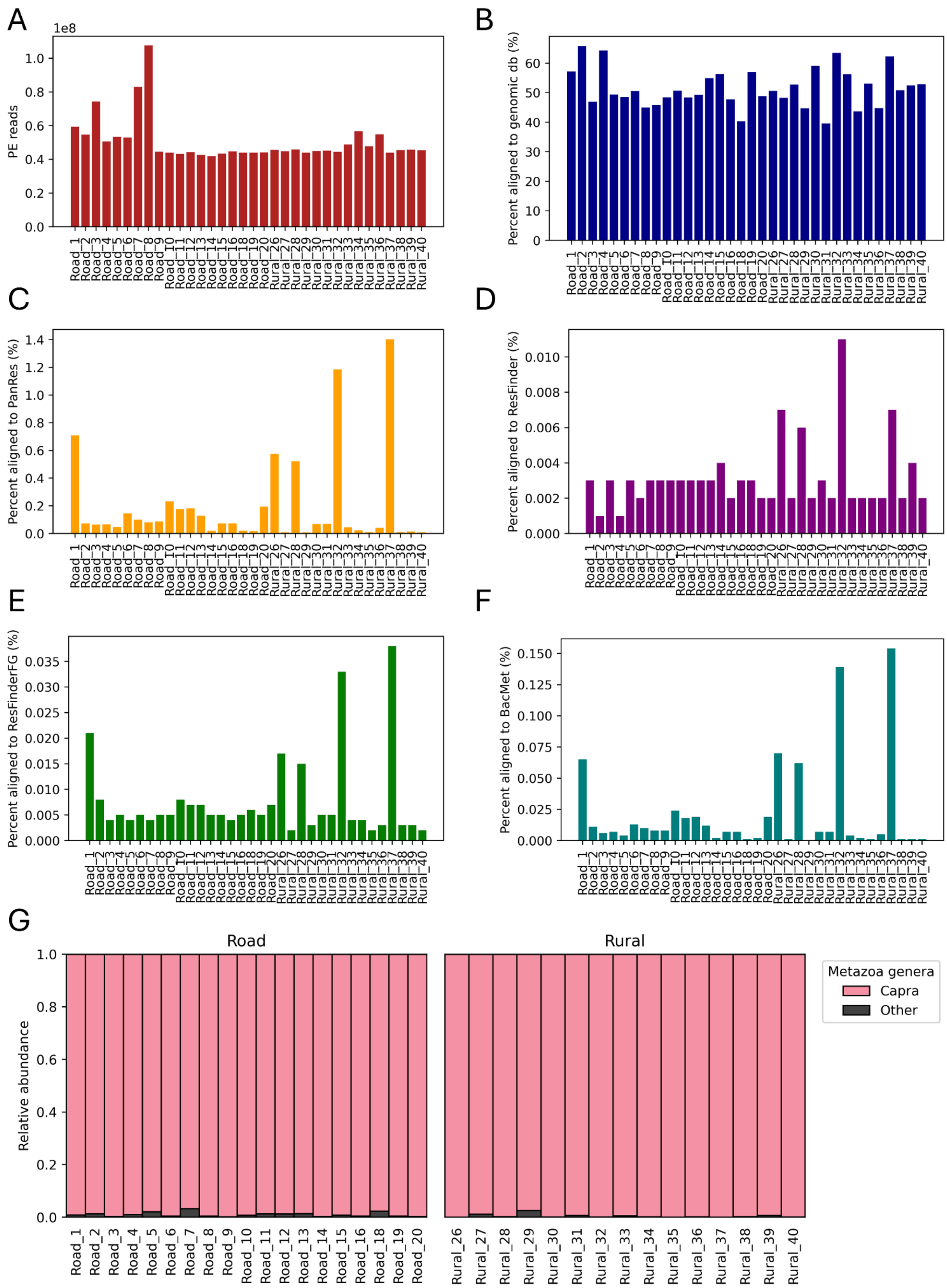

Figure S1: Overview of the 34 sequenced goat gut microbiome samples. **A)** Paired-end reads per sample. **B)** Percent of the total reads that were aligned to genomes from the custom genomic database consisting of the assembled, dereplicated MAGs, and bacterial genomes

and draft genomes from NCBI Genbank. **C)** Percent of the total reads that were mapped to the PanRes database. **D)** Percent of the total reads that were mapped to the ResFinder database (part of PanRes). **E)** Percent of the total reads that were mapped to the ResFinderFG database (part of PanRes). **F)** Percent of the total reads that were mapped to the BacMet database (part of PanRes). **G)** Relative abundance of the genera from the Metazoa kingdom, based on the read-mapping to the custom mitochondria database.

Supplementary figure S2

A

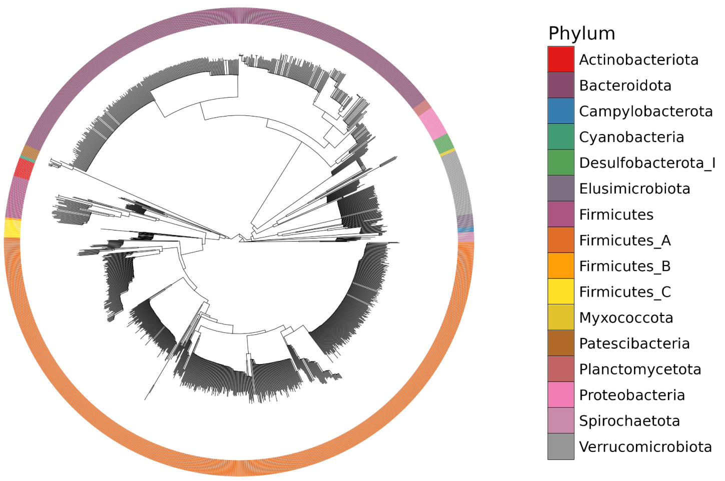

B

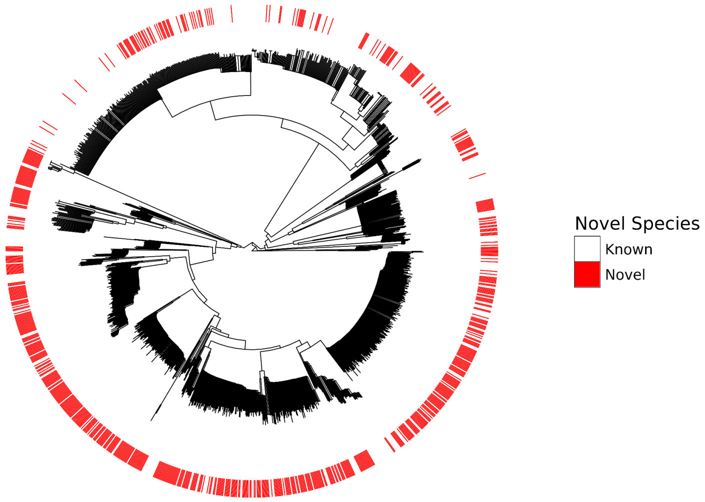

Figure S2: Taxonomy of the 1,468 dereplicated MAGs. **A)** Phylum level. **B)** Scattering of the unidentified/novel species (no GTDB-Tk annotation at the species level).

Supplementary table 2

Table S2: Top 50 most abundant bacterial species within the gut microbiome of Tanzanian goats calculated as centered log-ratios (clr).

**Bacterial species**	**Clr median**	**Clr variance**
Escherichia coli	12.890	3.726
Phocaeicola sp015060705	12.018	0.891
Phocaeicola sp017503235	11.513	1.687
Phocaeicola sp017416735	11.320	0.913
UBA2862 sp902765305	11.306	0.446
Cryptobacteroides sp017398335	11.216	7.073
CAG-83 sp017415775	10.768	1.930
Phascolarctobacterium_A sp017623055	10.621	15.691
Alistipes sp017399485	10.590	6.427
Cryptobacteroides sp017465225	10.522	3.240
GCA-900199385 sp902764875	10.408	0.620
Pradoshia eiseniae	10.277	2.022
Evtepia sp017521345	10.205	0.724
Alistipes sp015059845	10.143	16.211
HGM04593 sp015060965	10.043	4.262
Faecousia sp015067725	9.896	1.428
UBA1189 sp017622495	9.777	16.402
UBA11524 sp017936765	9.766	0.900
HGM04593 sp017467015	9.750	4.959
Cryptobacteroides sp017938925	9.710	1.714
Faecousia sp017436955	9.632	0.781
Limimorpha sp015061365	9.627	11.068
SIG307 sp017463035	9.594	5.374
Alistipes sp017403125	9.577	2.988
Copromorpha sp017416795	9.568	9.935
Alistipes sp017624955	9.559	26.684
SIG483 sp015068035	9.548	1.829
Alistipes sp017621455	9.542	5.168
CAG-83 sp017937195	9.538	1.230
RUG11797 sp015068345	9.524	5.156
Cryptobacteroides sp015060395	9.476	7.431
Akkermansia sp015061985	9.473	36.892
Amycolatopsis nivea	9.452	1.062
Fimenecus sp017399405	9.361	7.559
DTU089 sp017937865	9.340	10.143

UBA2862 sp900315585	9.308	0.625
Bacteroides xylanisolvens	9.297	2.563
UBA2862 sp017432405	9.248	0.727
Butyricimonas virosa	9.244	18.877
RUG472 sp902765005	9.223	0.820
UMGS1668 sp017521825	9.114	10.305
Clostridiales bacterium	9.063	0.880
Aliidongia dinghuensis	9.022	1.304
Egerieousia sp017935505	9.004	32.769
RGIG4079 sp017399915	8.983	3.402
Dysosmobacter sp. Marseille-Q4140	8.917	0.389
Pseudoflavitalea rhizosphaerae	8.902	1.122
Barnesiella_A sp017532725	8.884	23.390
RUG563 sp015056015	8.882	7.681
SFMI01 sp017532155	8.882	5.072

Supplementary table 3

Table S3: Top 20 most abundant bacterial genera within the gut microbiome of Tanzanian goats calculated as centered log-ratios (clr).

Genus	CLR median	CLR variance
Faecousia	12.489	0.145
Cryptobacteroides	12.481	1.064
Alistipes	12.245	0.902
Phocaeicola	11.876	0.386
UBA2862	11.556	0.675
Escherichia	11.507	4.335
CAG-83	11.329	1.819
UBA11524	10.992	0.267
HGM04593	10.974	1.629
Akkermansia	10.899	33.286
DTU089	10.770	5.989
UBA1189	10.265	1.964
SIG307	10.179	6.286
Ruminococcus_E	10.141	0.319
GCA-900199385	10.121	0.780
Limiplasma	10.093	0.877
Ruminococcus	9.978	1.753
Limimorpha	9.805	1.841
Barnesiella_A	9.625	1.426
Egerieousia	9.592	5.699

Supplementary figure 3

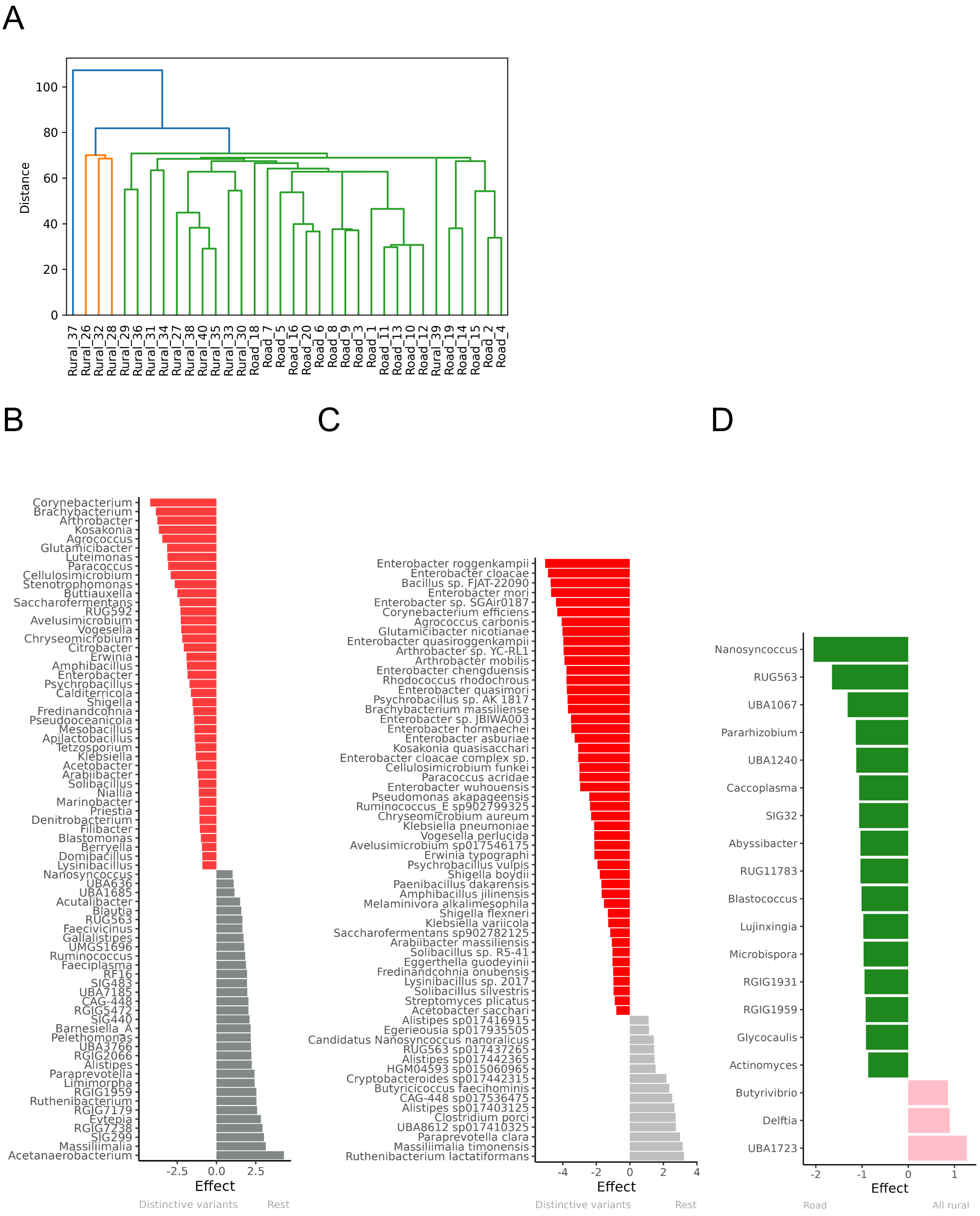

Figure S3: Unsupervised clustering analysis and differential abundance analysis. **A)** Hierarchy clustering on the clr-transformed genera counts. **B)** Differential abundance analysis on bacterial genera. Distinctive variants (Rural_26, Rural_28, Rural_32, and Rural_37) are colored red and the rest of the samples are colored grey. Statistically significant features with absolute effect size > 0.8 and FDR < 0.05 are shown. **C)** Differential abundance analysis on bacterial species. Distinctive variants (Rural_26, Rural_28, Rural_32, and Rural_37) are

colored red and the rest of the samples are colored grey. Statistically significant features with absolute effect size > 0.8 and FDR < 0.05 are shown. **D)** Differential abundance analysis: All road samples in green vs all rural samples in pink. Statistically significant features with absolute effect size > 0.8 and FDR < 0.05 are shown.

Supplementary figure 4

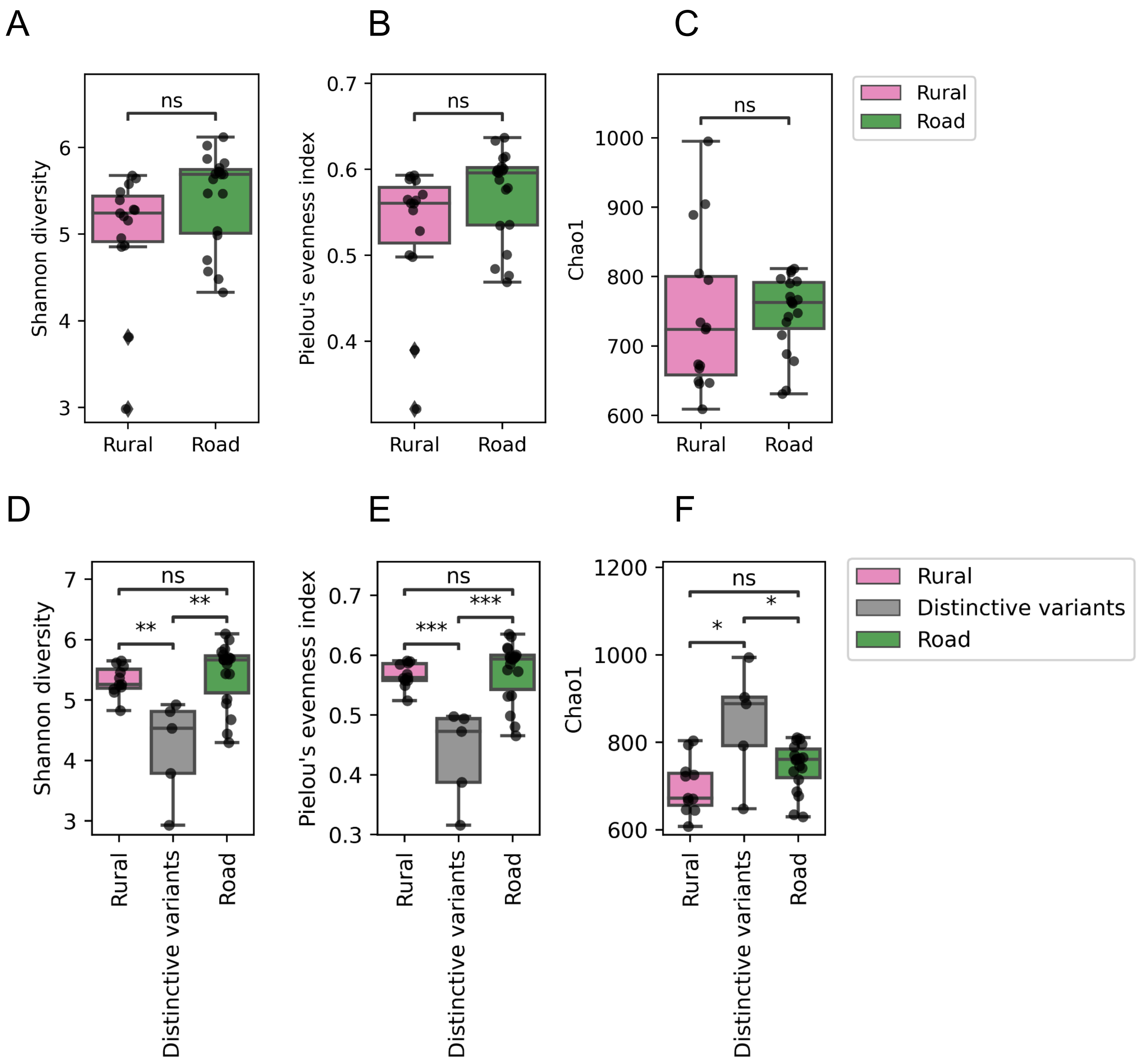

Figure S4: Bacterial diversity measures for the rural and road Tanzanian goats. Significance was tested with either an independent t-test or a Welch t-test if the assumption of equal variances was not met: *$P < 0.05$, **$P < 0.01$, ***$P < 0.001$. The distinctive variants were: Rural_26, Rural_28, Rural_32, and Rural_37 and are grouped as their own group in D-F. **A)** Bacterial genus Shannon diversity (P=9.0001e-02). **B)** Bacterial genus Pielou's evenness index (P=9.5543e-02). **C)** Bacterial genus Chao1 (P=8.7043e-01). **D)** Bacterial genus Shannon diversity (Rural vs Road, P=5.2524e-01; Rural vs Distinctive variants, P=3.8468e-02; Road vs Distinctive variants, P=5.5733e-04). **E)** Bacterial genus Pielou's evenness index (Rural vs Road, P=6.9950e-01; Rural vs Distinctive variants, P=1.8933e-02; Road vs Distinctive variants, P=8.6664e-05). **F)** Bacterial genus Chao1 (Rural vs Road, P=5.2445e-02; Rural vs Distinctive variants, P=8.0702e-03; Road vs Distinctive variants, P=1.5887e-02).

Supplementary table 4

Table S4: Top 20 most abundant resistance genes from ResFinder database within the gut microbiome of Tanzanian rural and road goats calculated as centered log-ratios on the length adjusted counts. All samples were included.

**Road**		
**Resistance gene from ResFinder db**	**CLR median**	**CLR variance**
tet(w)_5_aj427422	6.712	0.383
tet(q)_2_x58717	4.000	2.616
tet(o/32/o)_6_ng_048124	3.343	0.194
tet(44)_2_fn594949	2.974	0.387
tet(w)_2_ay049983	2.691	0.383
tet(44)_1_nz_abdu01000081	2.068	2.447
lnu(c)_1_ay928180	2.050	1.003
tet(x6)_1_mn507533	1.809	3.080
tet(w/32/o)_1_am710601	1.695	1.165
ant(6)-ia_3_kf864551	1.625	0.896
blaoxa-347_1_acwg01000053	1.516	3.466
msr(d)_2_af274302	1.380	0.476
cfr(c)_2_canb01000378	1.093	0.337
tet(w/32/o)_3_am710603	0.521	0.971
msr(d)_3_af227520	0.424	1.120
erm(f)_3_m17808	0.373	3.706
nimj_1_nz_jh815495	0.107	0.554
tet(o/w/o)-1_1_ay196921	0.107	0.481
tet(o)_2_m20925	0.107	0.258
tet(l)_1_hm235948	0.107	1.139

**Rural**		
**Resistance gene from ResFinder db**	**CLR median**	**CLR variance**
tet(w)_5_aj427422	6.403	1.075
tet(o/32/o)_6_ng_048124	3.764	1.589
sul2_2_ay034138	3.547	4.100
tet(w)_2_ay049983	3.068	2.247
aph(6)-id_1_m28829	2.841	3.358
lnu(c)_1_ay928180	2.738	1.891
qnrs1_1_ab187515	2.538	2.955
tet(x6)_1_mn507533	2.071	3.415
blaoxa-347_1_acwg01000053	1.789	3.276
erm(b)_12_u18931	1.679	4.311
sul2_9_fj197818	1.655	2.866
tet(44)_2_fn594949	1.595	0.737
ant(6)-ia_3_kf864551	1.307	3.006
tet(a)_6_af534183	1.267	2.109
erm(f)_3_m17808	1.235	4.277
tet(44)_1_nz_abdu01000081	1.095	1.551
cfr(c)_2_canb01000378	0.953	1.027
tet(l)_1_hm235948	0.719	2.393
sul3_2_aj459418	0.450	1.809
tet(b)_2_af326777	0.403	1.711

Supplementary figure 5

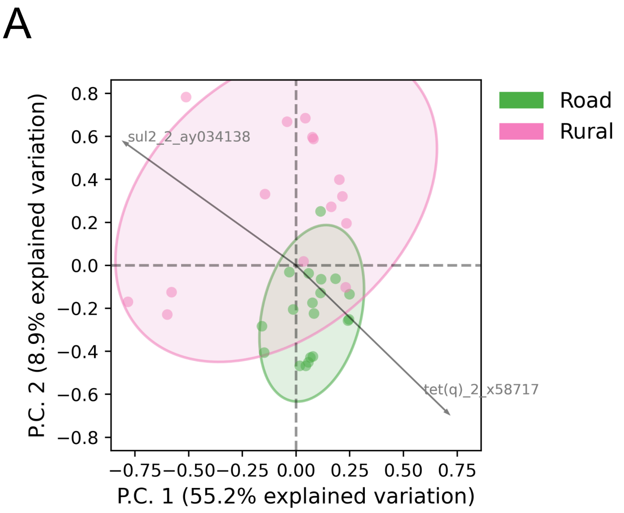

Figure S5: Resistome difference in road rural Tanzanian goats based on read-mapping to the ResFinder database. **A)** Clustering of AMR genes from the ResFinder database. Only *sul2_2_ay034138* which drives the rural goats (effect = -1.460) and *tet(q)_2_x58717* which drives the road goats (effect=1.134) are shown. **B)** Total AMR load calculated as FPKM (Welch t-test, P=0.034). *$P < 0.05$, **$P < 0.01$, ***$P < 0.001$. The four distinct variants had a higher AMR load: Rural_32 (FPKM=307), Rural_26 (FPKM=240), Rural_28 (FPKM=224), and Rural_37 (FPKM=160).

Supplementary figure 6

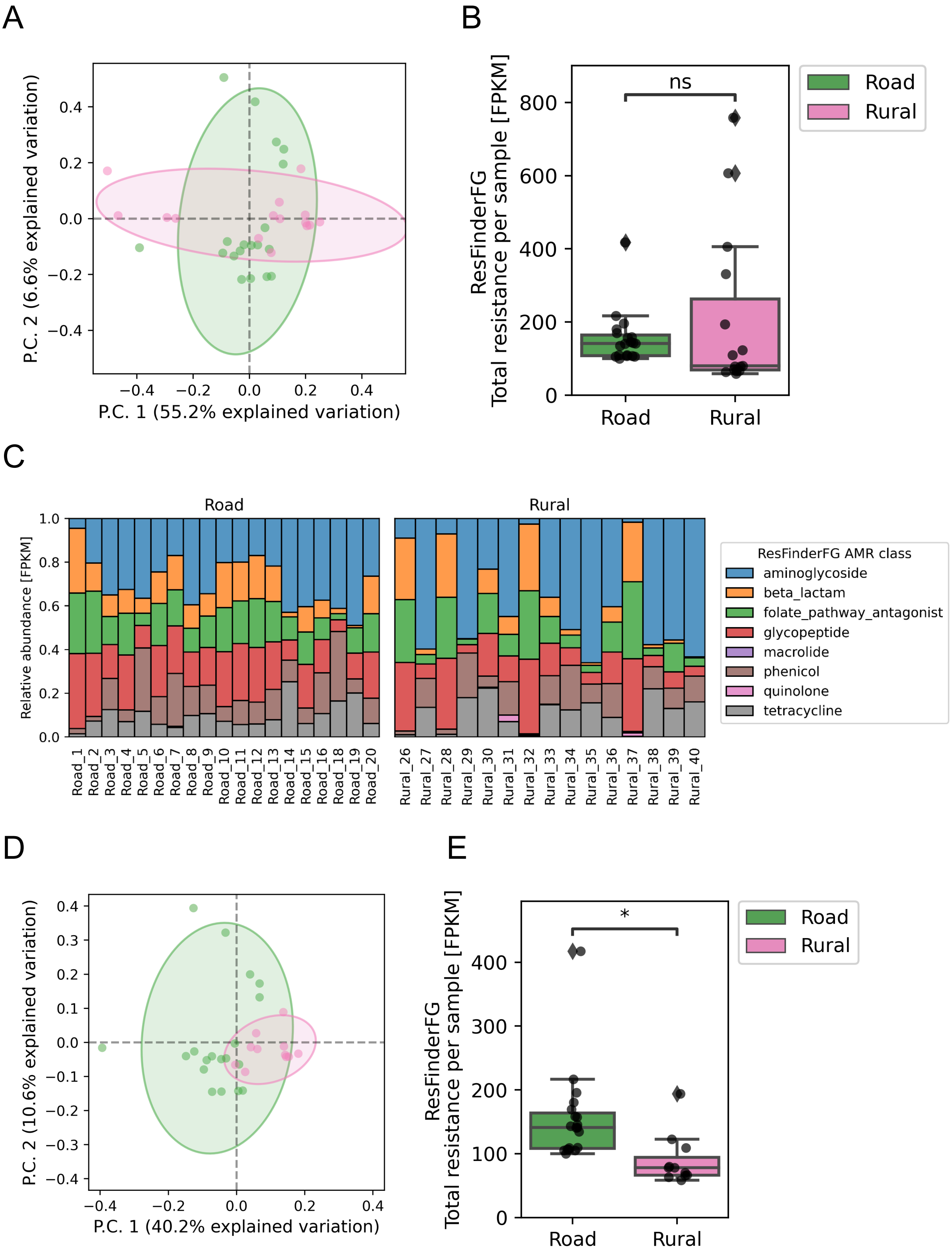

Figure S6: Resistome differences in road and rural Tanzanian goats based on read-mapping to the ResFinderFG database. **A)** Clustering of AMR genes from the ResFinderFG database. No differential abundant genes were detected. **B)** Total AMR load calculated as FPKM (independent t-test, *P*=3.513e-01). **C)** Relative abundance of the resistome for road and rural

goats calculated as FPKM. **D)** Clustering of AMR genes from the ResFinderFG database. The four distinct variants were not included (Rural_32, Rural_26, Rural_28 and Rural_37). No differential abundant genes were detected. **E)** Total resistance load calculated as FPKM (independent t-test independent, *P*=1.0032e-02). The four distinct variants were not included (Rural_26, Rural_28, Rural_32, and Rural_37).

Supplementary table 5

Table S5: Top 20 most abundant AMR genes from ResFinderFG database within the gut microbiome of Tanzanian all rural and road goats calculated as centered log-ratios on the length adjusted counts.

**Road**		
**Resistance gene from ResFinderFG**	**CLR median**	**CLR variance**
functional_amr|dfr|kx126113.1|newborn_gut|sxt	5.574	0.966
functional_amr|aac|kj512951.1|manure|kan	5.571	0.415
functional_amr|van-ligase|kf628564.1|pediatric_fecal_sample|cyc	5.514	0.993
functional_amr|non-enz-beta-lactam-resistance|ku605919.1|preterm_infant_stool|pen	5.405	1.041
functional_amr|van-ligase|kf628791.1|pediatric_fecal_sample|cyc	5.274	0.999
functional_amr|cat|kj512930.1|manure|chl	4.442	0.391
functional_amr|tet-protection|kx126991.1|newborn_gut|tet	4.083	3.866
functional_amr|non-enz-beta-lactam-resistance|ku606326.1|preterm_infant_stool|amc	4.073	1.329
functional_amr|aac|kj512956.1|manure|kan	4.011	0.466
functional_amr|tet-protection|kx126524.1|newborn_gut|tet	3.956	1.061
functional_amr|cat|kj512920.1|manure|chl	3.925	0.712
functional_amr|bifunctional-aac/aph|kj512971.1|manure|kan	3.850	0.715
functional_amr|van-ligase|kf629112.1|pediatric_fecal_sample|cyc	3.699	0.479
functional_amr|cat|kj512912.1|manure|chl	3.634	0.907
functional_amr|cat|kj512927.1|manure|chl	3.479	0.894
functional_amr|aac|kj512958.1|manure|kan	3.473	0.985

**Rural**		
**Resistance gene from ResFinderFG**	**CLR median**	**CLR variance**
functional_amr|aac|kj512951.1|manure|kan	5.608	2.358
functional_amr|dfr|kx126113.1|newborn_gut|sxt	5.441	2.697
functional_amr|van-ligase|kf628791.1|pediatric_fecal_sample|cyc	5.149	3.241
functional_amr|non-enz-beta-lactam-resistance|ku605919.1|preterm_infant_stool|pen	5.046	3.673
functional_amr|van-ligase|kf628564.1|pediatric_fecal_sample|cyc	4.715	3.364
functional_amr|cat|kj512930.1|manure|chl	4.291	1.062
functional_amr|bifunctional-aac/aph|kj512971.1|manure|kan	4.285	0.666
functional_amr|tet-protection|kx126991.1|newborn_gut|tet	4.253	1.943
functional_amr|aac|kj512958.1|manure|kan	4.242	1.138
functional_amr|van-ligase|kf629112.1|pediatric_fecal_sample|cyc	4.087	1.139
functional_amr|beta-lactamase|ku547860.1|latrine|amx	3.994	1.944
functional_amr|tet-protection|kx126524.1|newborn_gut|tet	3.966	5.986
functional_amr|non-enz-beta-lactam-resistance|ku606326.1|preterm_infant_stool|amc	3.839	2.256
functional_amr|van-ligase|kx127228.1|newborn_gut|cyc	3.756	3.156
functional_amr|cat|kj512920.1|manure|chl	3.614	1.246
functional_amr|tet-protection|kf626853.1|pediatric_fecal_sample|tet	3.526	1.534

functional_amr|van-ligase|kf627523.1|pediatric_fecal_sample|cyc	3.413	0.288
functional_amr|cat|kj512915.1|manure|chl	3.370	0.418
functional_amr|tet-protection|kf626853.1|pediatric_fecal_sample|tet	3.305	0.963
functional_amr|van-ligase|kx128227.1|newborn_gut|cyc	3.240	0.825

functional_amr|cat|kj512912.1|manure|chl	3.337	3.413
functional_amr|cat|kj512927.1|manure|chl	3.152	1.506
functional_amr|aac|kj512956.1|manure|kan	2.992	1.128
functional_amr|tet-protection|ku546032.1|feces|tet	2.896	5.680
