# Supplementary material for: Tanzanian goat gut microbiomes adapt to roadside pollutants and environmental stressors
Source: Microbiol Spectr. 2025 Dec 17;14(2):e02036-25. doi: 10.1128/spectrum.02036-25 (PMC12889105; doi:10.1128/spectrum.02036-25)
Supplement: Supplemental material 2 — Tables S6 to S9; Fig. S7 to S12. [file spectrum.02036-25-s0002.pdf]

Supplementary material part 2

Supplementary figure 7

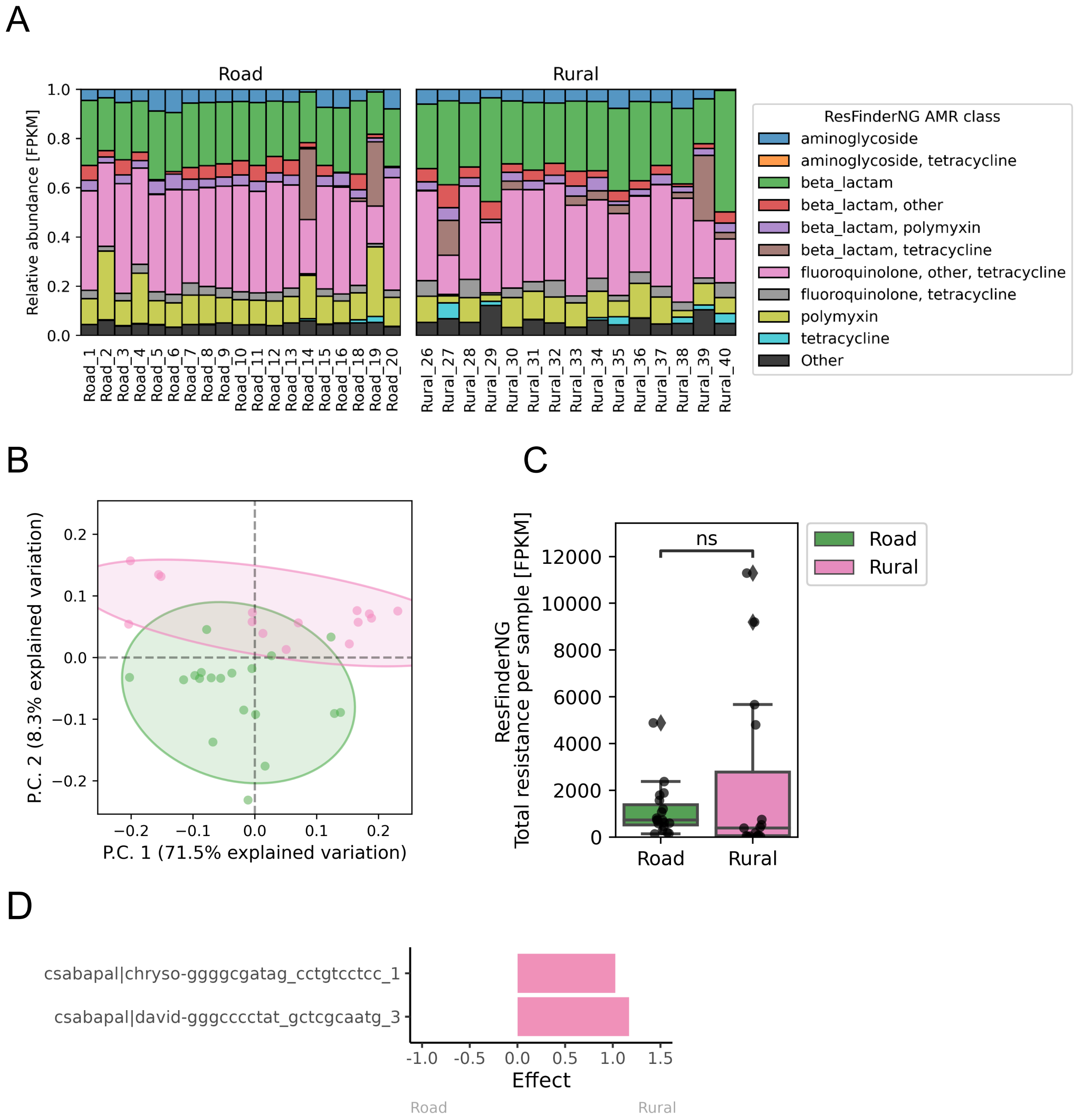

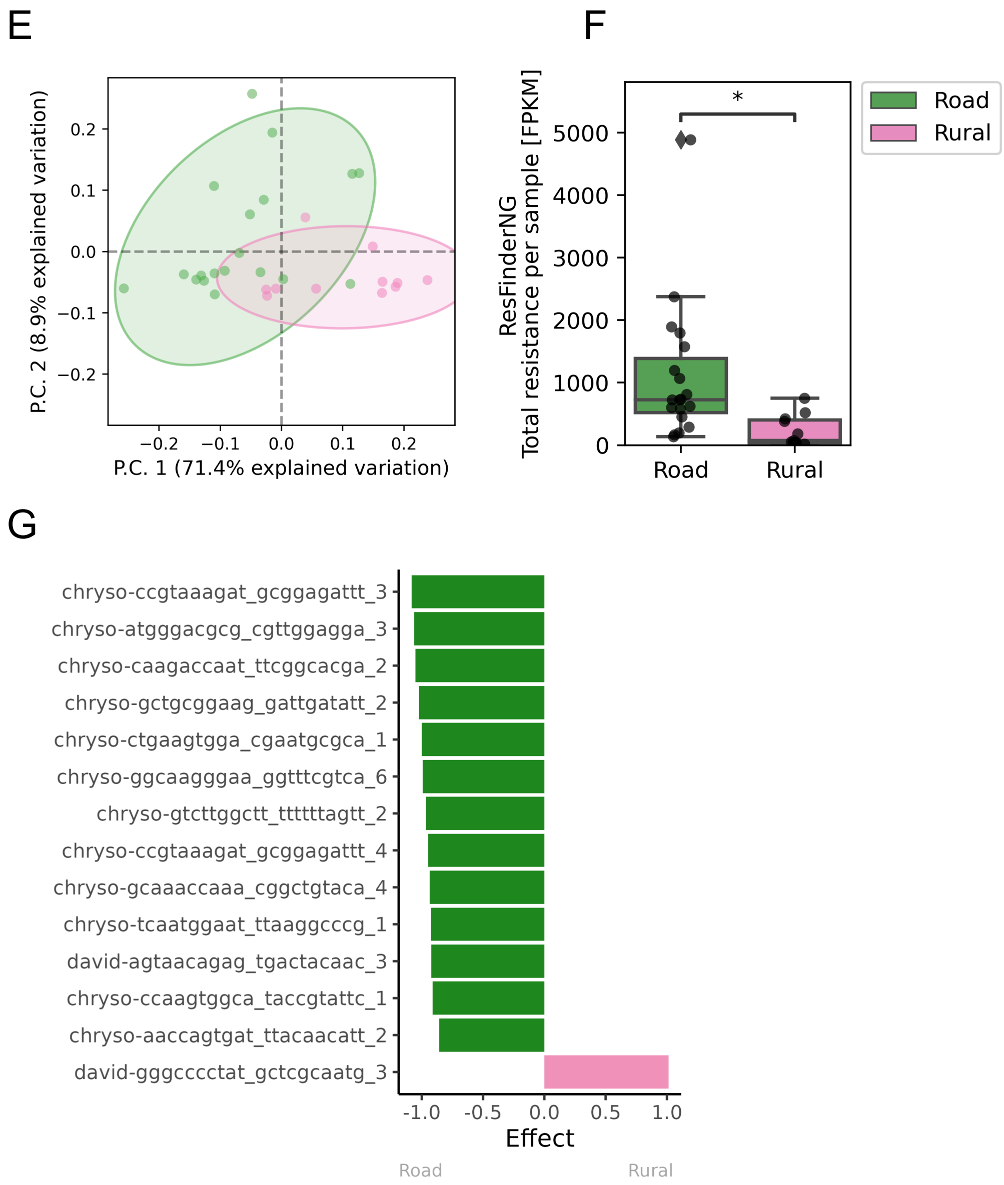

Figure S7: Resistome differences in road and rural Tanzanian goats based on read-mapping to the ResFinderNG database. **A)** Relative abundance of the resistome for road and rural goats calculated as FPKM. **B)** Clustering of AMR genes from the ResFinderNG database. **C)** Total AMR load calculated as FPKM (independent t-test independent, *P*=2.1541e-01). **D)** Differential abundant novel resistance genes driving the two groups (rural and road goats). Two genes were found to be differential abundant and driving the rural goats. **E)** Clustering of AMR genes from the ResFinderNG database. The four distinct variants were not included (Rural_32, Rural_26, Rural_28 and Rural_37). **F)** Total resistance load calculated as FPKM (independent t-test, *P*=1.704e-02). The four distinct variants were not included (Rural_26, Rural_28, Rural_32, and Rural_37). **G)** Differential abundant novel resistance genes driving the two groups. The four distinct variants were not included (Rural_32, Rural_26, Rural_28 and Rural_37).

S**upplementary table 6**

Table S6: Top 20 most abundant AMR genes from ResFinderNG database within the gut microbiome of Tanzanian all rural and road goats calculated as centered log-ratios on the length adjusted counts.

Road		
**Resistance gene from the ResFinderNG db**	**CLR median**	**CLR variance**
chryso-ccgtaaagat_gcggagattt_3	5.179	0.246
chryso-ccgtaaagat_gcggagattt_4	4.999	0.370
chryso-tcaatggaat_ttaaggcccg_1	4.995	0.357
chryso-aatgaacaga_ttgcaggatc_2	4.987	1.377
chryso-gtcttggctt_ttttttagtt_3	4.963	0.225
chryso-caagaccaat_ttcggcacga_3	4.943	0.207
chryso-atgggacgcg_cgttggagga_3	4.905	0.288
chryso-ctgaagtgga_cgaatgcgca_1	4.894	0.201
david-agtaacagag_tgactacaac_3	4.825	0.303
chryso-gagcgtcgac_tttaacacag_2	4.816	0.205
chryso-aaccggaagc_acctcattcg_5	4.807	0.150
chryso-gtcttggctt_ttttttagtt_2	4.802	0.386
chryso-ggtagtaaga_tgctaccgag_2	4.783	0.393
chryso-aaacaggaat_ttccactgat_2	4.778	0.466
chryso-caagaccaat_ttcggcacga_2	4.749	0.187
chryso-aaccggaagc_acctcattcg_4	4.746	0.316
chryso-aaccagtgat_ttacaacatt_2	4.739	0.219
chryso-aaccggaagc_acctcattcg_3	4.704	1.843
chryso-ggcaagggaa_ggtttcgtca_6	4.676	0.167
chryso-aaacaggaat_ttccactgat_1	4.673	0.302

Rural		
**Resistance gene from the ResFinderNG db**	**CLR median**	**CLR variance**
chryso-tcaatggaat_ttaaggcccg_1	4.558	0.266
chryso-ccgtaaagat_gcggagattt_3	4.529	0.207
chryso-ggtagtaaga_tgctaccgag_2	4.497	0.520
chryso-caagaccaat_ttcggcacga_3	4.487	0.397
chryso-gtcttggctt_ttttttagtt_3	4.436	0.468
chryso-ccgtaaagat_gcggagattt_4	4.425	0.137
chryso-gagcgtcgac_tttaacacag_2	4.383	0.391
david-agtaacagag_tgactacaac_3	4.375	0.441
chryso-aatgaacaga_ttgcaggatc_2	4.341	0.528
chryso-aaccagtgat_ttacaacatt_2	4.341	0.256
chryso-aaacaggaat_ttccactgat_2	4.331	0.795
chryso-ctgaagtgga_cgaatgcgca_1	4.330	0.496
chryso-aaccggaagc_acctcattcg_3	4.326	1.195
chryso-atgggacgcg_cgttggagga_3	4.326	0.315
chryso-aaccggaagc_acctcattcg_5	4.307	0.466
chryso-aaccggaagc_acctcattcg_4	4.299	0.333
chryso-gtcttggctt_ttttttagtt_2	4.259	0.117
chryso-caagaccaat_ttcggcacga_2	4.244	0.197
chryso-tcaatggaat_ttaaggcccg_2	4.206	0.698
chryso-aaacaggaat_ttccactgat_1	4.177	0.676

Supplementary figure 8

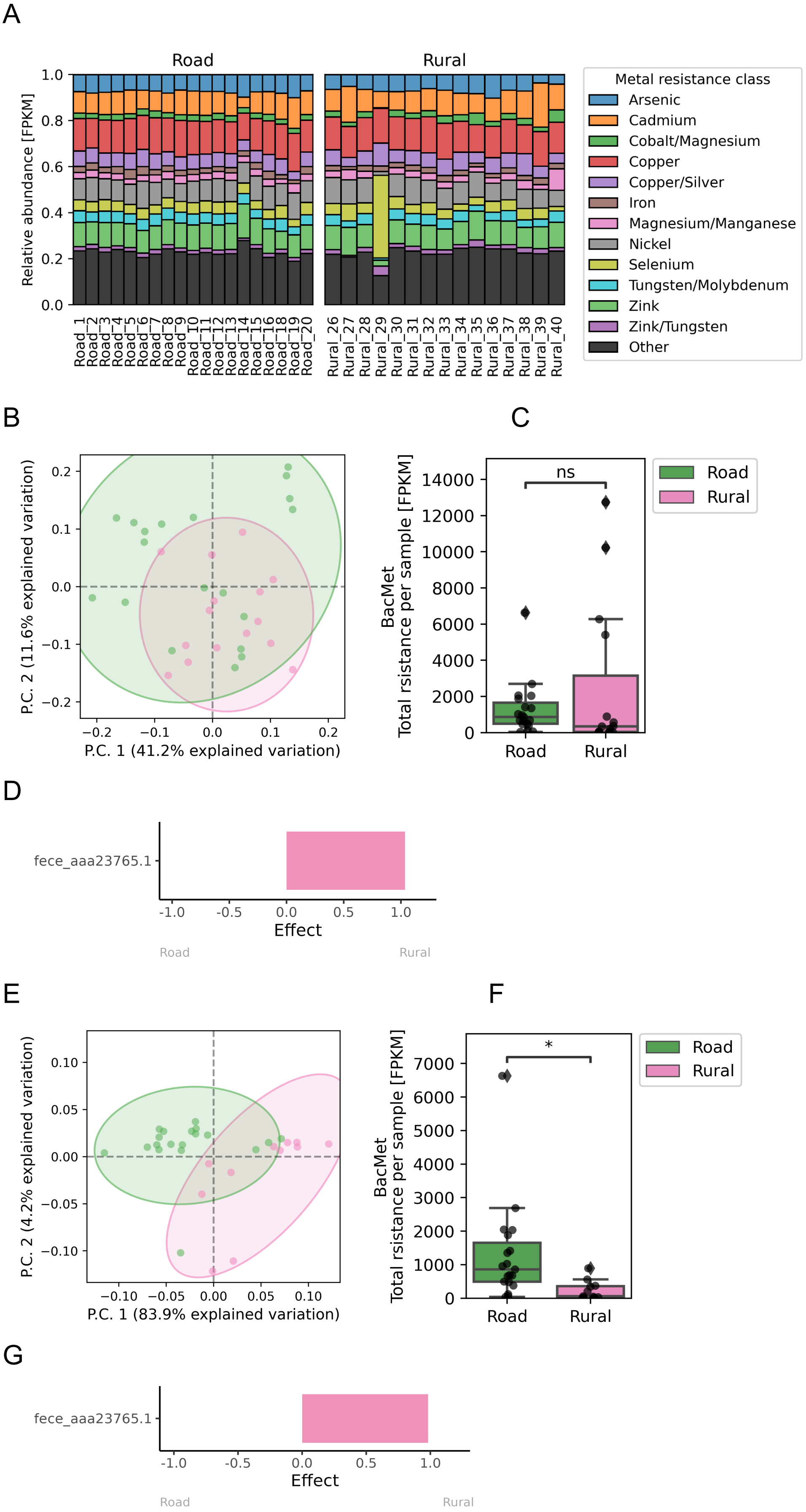

Figure S8: Metal resistance differences in road and rural Tanzanian goats based on read-mapping to the BacMet database. **A)** Relative abundance of metal resistance for road and rural goats calculated as FPKM. **B)** Clustering of metal resistance genes from the BacMet database. Only features with clr median > 0 were included. **C)** Total metal resistance load calculated as FPKM (independent t-test, *P*=2.5626e-01). **D)** Differential abundant metal resistance genes. **E)** Clustering of metal resistance genes from BacMet database. The four distinct variants were not included (Rural_26, Rural_28, Rural_32, and Rural_37). **F)** Total metal resistance load calculated as FPKM (independent t-test, *P*=2.8510e-02) with the four distinctive microbiomes removed (Rural_32, Rural_26, Rural_28 and Rural_37). **G)** Differential abundant metal resistance genes. The four distinct variants were not included (Rural_26, Rural_28, Rural_32, and Rural_37).

Supplementary table 7

Table S7: Top 20 most abundant metal resistance genes from BacMet database within the gut microbiome of all Tanzanian rural and road goats calculated as centered log-ratios on the length adjusted counts.

Road		
**Metal resistance gene**	**CLR median**	**CLR variance**
nikb_bae77816.1	4.114	0.018
nika_bae77817.1	4.108	0.016
znta/yhho_bae77824.1	4.099	0.028
nikc_bae77815.1	4.063	0.142
zrar/hydh_bae77315.1	4.048	0.029
nikd_bae77814.1	3.997	0.014
cusa/ybde_baa35215.1	3.980	0.018
yqjh_bae77120.1	3.970	0.012
mgta_bae78241.1	3.934	0.009
cusb_baa35208.1	3.922	0.019
glpf_bae77383.1	3.901	0.061
pita_bae77801.1	3.889	0.022
psta_bae77562.1	3.887	0.021
cute/lnt_baa35308.2	3.886	0.008
fief/yiip_bae77395.1	3.885	0.018
cora_bae77485.1	3.881	0.142
cueo_bab96698.2	3.877	0.029
pstc_bae77561.1	3.868	0.027
moda_baa35427.1	3.868	0.018
roba_bae78385.1	3.862	0.029

Rural		
**Metal resistance gene**	**CLR median**	**CLR variance**
nikb_bae77816.1	3.386	0.114
nika_bae77817.1	3.379	0.102
znta/yhho_bae77824.1	3.355	0.142
nikd_bae77814.1	3.324	0.262
zrar/hydh_bae77315.1	3.293	0.029
nikc_bae77815.1	3.290	0.236
cusa/ybde_baa35215.1	3.290	0.034
fief/yiip_bae77395.1	3.254	0.130
cusb_baa35208.1	3.233	0.141
mgta_bae78241.1	3.231	0.085
yqjh_bae77120.1	3.222	0.152
nike_bae77813.1	3.206	0.229
roba_bae78385.1	3.204	0.232
cueo_bab96698.2	3.202	0.110
cora_bae77485.1	3.181	0.845
cusr/ylca_baa35205.1	3.180	0.297
dsbc_bae76958.1	3.169	0.555
baer_baa15935.1	3.150	0.096
cute/lnt_baa35308.2	3.149	0.048
cuss_baa35204.2	3.131	0.066

Supplementary table 8

Table S8: Top 10 most abundant plastic types that enzymes were able to degrade from the collection of enzymes from plasticDB within the gut microbiome of all Tanzanian rural and road goats. The values are calculated as centered log-ratios (clr) on the length adjusted counts.

Road		
**Plastic type**	**CLR median**	**CLR variance**
PBS_PBSA_PCL	3.053	0.300
P3HV_PHBV_PHA	2.557	0.045
PEG	2.447	0.259
PLA	2.195	0.065
PLA_PBSA	2.044	0.032
PBS_PBSA_PCL_PES_PHB_PLA_PHA	1.965	0.243
PBAT	1.868	0.030
PBSA	1.861	0.217
PHB_PHA	1.716	0.335
PU	1.708	0.023

Rural		
**Plastic type**	**CLR median**	**CLR variance**
P3HV_PHBV_PHA	3.110	0.110
PLA	2.957	0.187
PEG	2.662	0.134
PBSA	2.486	0.320
PLA_PBSA	2.482	0.160
PBS_PBSA_PCL	2.438	0.262
PBS_PBSA_PCL_PES_PHB_PLA_PHA	2.372	0.219
PHB_PHA	2.351	0.136
PBAT	2.344	0.046
PU	2.262	0.176

Supplementary table 9

Table S9: Top 20 most abundant enzymes with plastic-degrading capabilities from the collection of enzymes from plasticDB within the gut microbiome of all Tanzanian rural and road goats. The values are calculated as centered log-ratios (clr) on the length adjusted counts.

Road		
**Enzyme with capability of plastic biodegradation**	**CLR median**	**CLR variance**
00087||Polyesterase||Pseudomonas_pseudoalcaligenes||PBAT	4.525	3.549
00032||PEG_dehydrogenase||Sphingomonas_macrogoltabidus||PEG	4.157	1.711
00218||Chitinase||Geomyces_sp.||PBS_PBSA_PCL	4.015	0.481
00071||3HV_dehydrogenase||Paracoccus_denitrificans||P3HV_PHBV_PHA	3.879	0.167
00152||Protease||Lederbergia_lenta||PLA	3.604	0.445
00153||Protease||Lederbergia_lenta||PLA	3.594	0.441
00035||PEG_aldehyde_dehydrogenase||Streptomyces_sp.||PEG	3.563	0.110
00050||PLA_depolymerase||Uncultured_bacterium||PLA	3.377	0.223
00077||Esterase||Clostridium_botulinum||PBAT	3.362	0.203
00080||Lipase||Pelosinus_fermentans||PBAT	3.348	0.183
00049||PLA_depolymerase||Uncultured_bacterium||PBS_PBSA_PCL_PES_PHB_PLA_PHA	3.334	0.235
00078||Esterase||Clostridium_botulinum||PBAT	3.325	0.253
00154||Protease||Bacillus_licheniformis||PLA	3.320	0.227
00157||Esterase||Alcanivorax_borkumensis||PLA_PBSA	3.278	0.182
00089||Carboxylesterase||Uncultured_bacterium||PBAT	3.205	0.233
00056||Lipase||Burkholderia_cepacia||PBSA	3.192	0.409

Rural		
**Enzyme with capability of plastic biodegradation**	**CLR median**	**CLR variance**
00152||Protease||Lederbergia_lenta||PLA	3.924	0.500
00153||Protease||Lederbergia_lenta||PLA	3.918	0.482
00071||3HV_dehydrogenase||Paracoccus_denitrificans||P3HV_PHBV_PHA	3.872	0.224
00154||Protease||Bacillus_licheniformis||PLA	3.528	0.243
00087||Polyesterase||Pseudomonas_pseudoalcaligenes||PBAT	3.301	3.336
00035||PEG_aldehyde_dehydrogenase||Streptomyces_sp.||PEG	3.291	0.112
00157||Esterase||Alcanivorax_borkumensis||PLA_PBSA	3.262	0.311
00072||PHB_depolymerase||Bacillus_thuringiensis||PHB_PHA	3.188	0.327
00056||Lipase||Burkholderia_cepacia||PBSA	3.178	0.460
00218||Chitinase||Geomyces_sp.||PBS_PBSA_PCL	3.169	0.320
00185||PETase||Uncultured_bacterium||PET	3.088	0.321
00013||PU_esterase_||Comamonas_acidovorans||PU	3.064	0.339
00049||PLA_depolymerase||Uncultured_bacterium||PBS_PBSA_PCL_PES_PHB_PLA_PHA	3.057	0.219
00079||Esterase||Clostridium_hathewayi||PBAT	3.051	0.319
00078||Esterase||Clostridium_botulinum||PBAT	3.027	0.287
00048||PLA_depolymerase||Uncultured_bacterium||PBS_PBSA_PCL_PES_PLA_PHA	3.012	0.302

00072||PHB_depolymerase||Bacillus_thuringiensis||PHB_PHA	3.153	0.301
00086||Carboxylesterase||Uncultured_bacterium||PBAT	3.116	0.227
00158||Esterase||Uncultured_bacterium||PLA	3.110	0.300
00091||Carboxylesterase||Uncultured_bacterium||PBAT	3.095	0.368

00086||Carboxylesterase||Uncultured_bacterium||PBAT	3.010	0.340
00085||Carboxylesterase||Uncultured_bacterium||PBAT	3.009	0.212
00089||Carboxylesterase||Uncultured_bacterium||PBAT	2.976	0.335
00077||Esterase||Clostridium_botulinum||PBAT	2.966	0.163

Supplementary figure 9

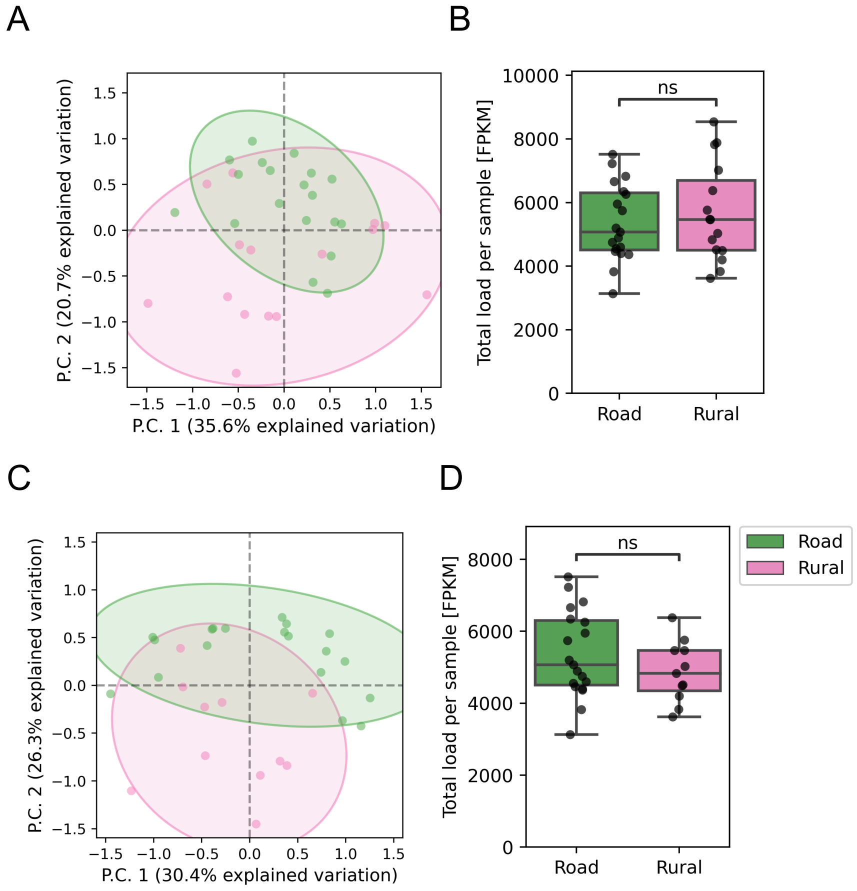

Figure S9: Differences in enzymes capable of plastic biodegradation. **A)** Clustering of all samples based on the plastic degrading enzymes (length-adjusted abundances). Differential abundance analysis revealed no enzymes being differential abundant between the two groups. **B)** Total plastic degradation calculated as FPKM (independent t-test*P*=5.2955e-01). **C)** Clustering of all samples excluding the distinctive variants (Rural_32, Rural_26, Rural_28 and Rural_37) based on the plastic degrading enzymes (length-adjusted abundances). Differential abundance analysis revealed no enzymes being differential abundant between the two groups. **D)** Total plastic degradation calculated as FPKM. The four distinct variants were not included (Rural_32, Rural_26, Rural_28 and Rural_37) (independent t-test, *P*=2.5101e-01).

Supplementary figure 10

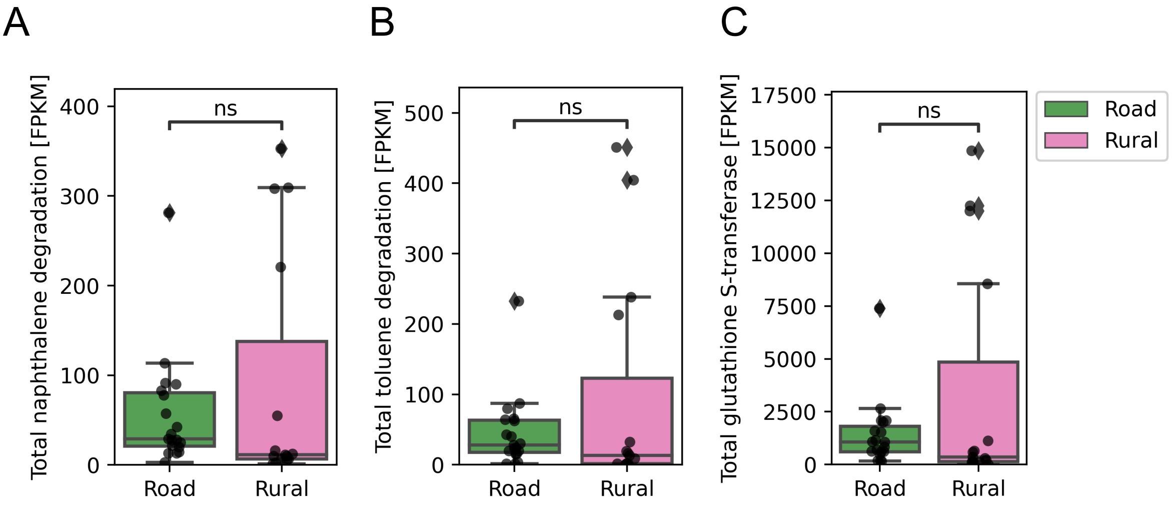

Figure S10: Differences in the total naphthalene and toluene degradation as well as total glutathione S-transferase in all samples. **A)** Total naphthalene degradation calculated as FPKM in road and rural Tanzanian goats (independent t-test independent, *P*=0.3.5617e-01. **B)** Total toluene degradation calculated as FPKM in road and rural Tanzanian goats (independent t-test, *P*=3.5617e-01). **C)** Total glutathione S-transferase calculated as FPKM in road and rural Tanzanian goats (independent t-test, *P*=3.5617e-01).

Supplementary figure 11

A

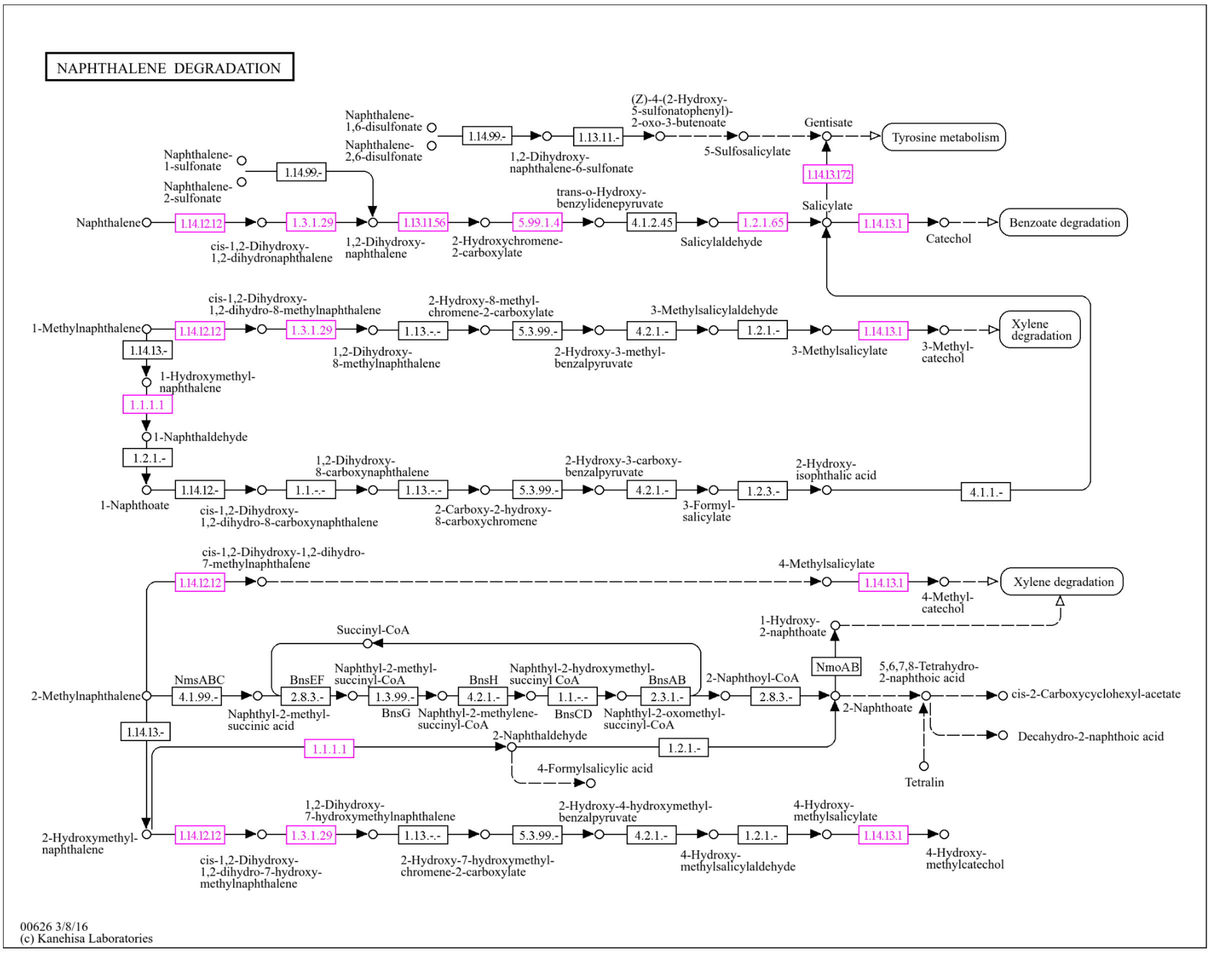

B

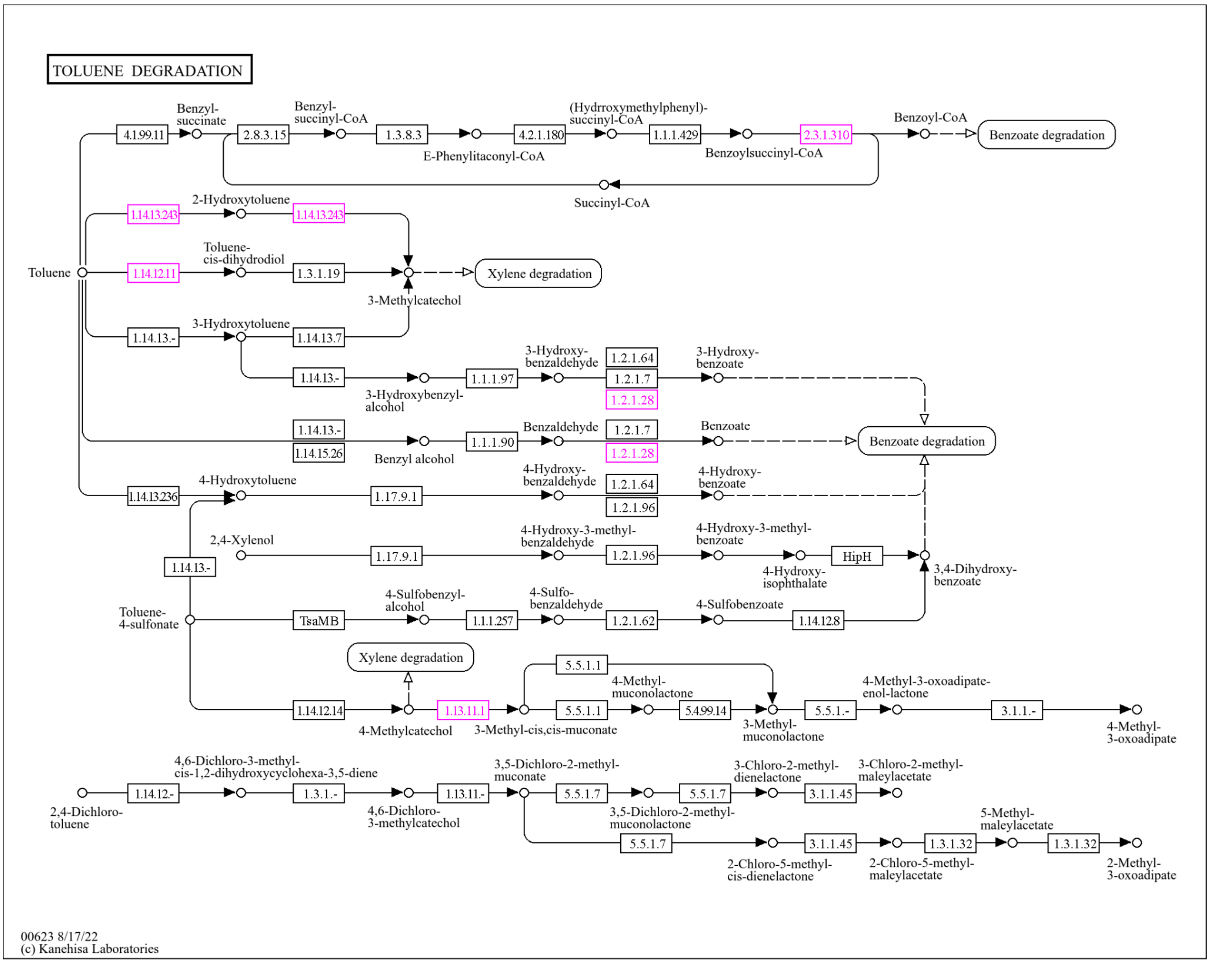
TOLUENE DEGRADATION
Toluene
Benzyl-succinate
Benzyl-succinyl-CoA
E-Phenylitaconyl-CoA
(Hydrroxymethylphenyl)-succinyl-CoA
Benzoylsuccinyl-CoA
Benzoyl-CoA
Succinyl-CoA
Benzoate degradation
4.1.99.11
2.8.3.15
1.3.8.3
4.2.1.180
1.1.1.429
2.3.1.310
2-Hydroxytoluene
1.14.13.243
Toluene-cis-dihydrodiol
1.14.12.11
1.3.1.19
3-Methylcatechol
Xylene degradation
3-Hydroxytoluene
1.14.13.-
1.14.13.7
3-Hydroxybenzyl-alcohol
1.1.1.97
3-Hydroxy-benzaldehyde
1.2.1.64
1.2.1.7
1.2.1.28
3-Hydroxy-benzoate
1.14.15.26
Benzyl alcohol
1.1.1.90
Benzaldehyde
Benzoate
4-Hydroxytoluene
1.14.13.236
1.17.9.1
4-Hydroxy-benzaldehyde
1.2.1.96
4-Hydroxy-benzoate
2,4-Xylenol
4-Hydroxy-3-methyl-benzaldehyde
4-Hydroxy-3-methyl-benzoate
4-Hydroxy-isophthalate
HipH
3,4-Dihydroxy-benzoate
Toluene-4-sulfonate
TsaMB
4-Sulfobenzyl-alcohol
1.1.1.257
4-Sulfo-benzaldehyde
1.2.1.62
4-Sulfobenzoate
1.14.12.8
1.14.12.14
4-Methylcatechol
1.13.11.1
3-Methyl-cis,cis-muconate
5.5.1.1
4-Methyl-muconolactone
5.4.99.14
3-Methyl-muconolactone
5.5.1.-
4-Methyl-3-oxoadipate-enol-lactone
3.1.1.-
4-Methyl-3-oxoadipate
2,4-Dichloro-toluene
1.14.12.-
4,6-Dichloro-3-methyl-cis-1,2-dihydroxycyclohexa-3,5-diene
1.3.1.-
4,6-Dichloro-3-methylcatechol
1.13.11.-
3,5-Dichloro-2-methyl-muconate
5.5.1.7
3-Chloro-2-methyl-dienelactone
3.1.1.45
3-Chloro-2-methyl-maleylacetate
3,5-Dichloro-2-methyl-muconolactone
2-Chloro-5-methyl-cis-dienelactone
2-Chloro-5-methyl-maleylacetate
1.3.1.32
5-Methyl-maleylacetate
2-Methyl-3-oxoadipate
00623 8/17/22
(c) Kanehisa Laboratories

C

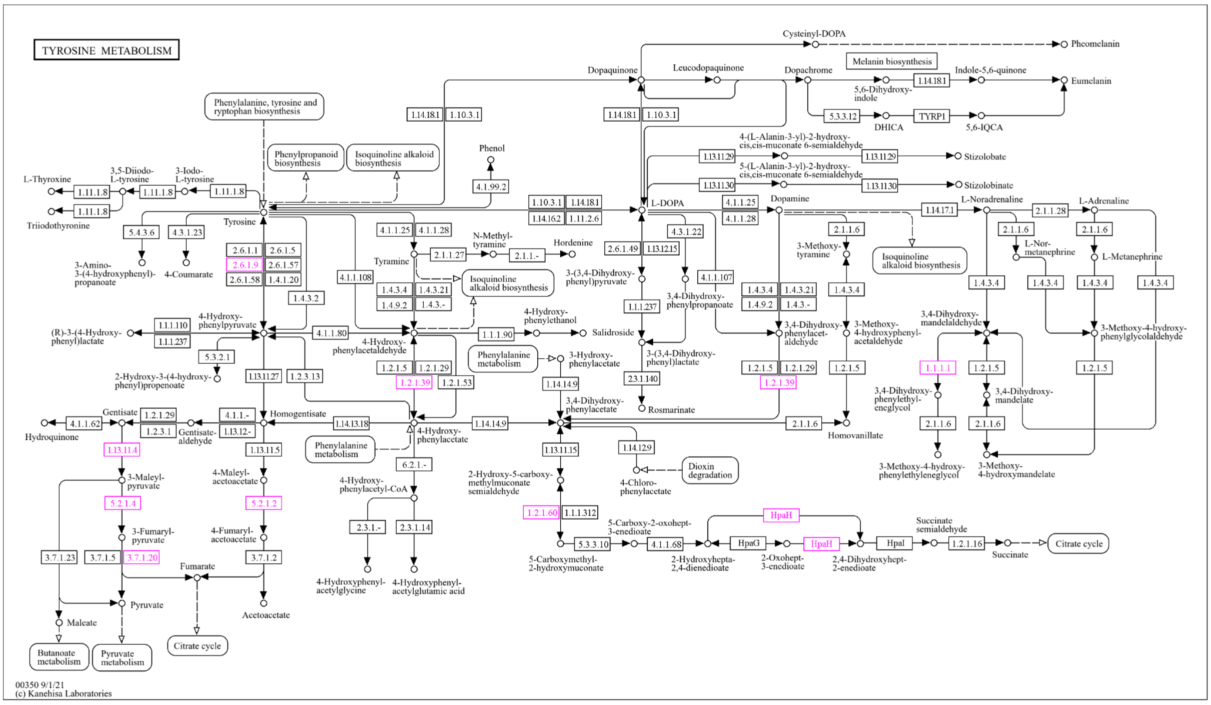
TYROSINE METABOLISM
Phenylalanine, tyrosine and tryptophan biosynthesis
Phenylpropanoid biosynthesis
Isoquinoline alkaloid biosynthesis
L-Thyroxine
Triiodothyronine
3,5-Diiodo-L-tyrosine
3-Iodo-L-tyrosine
Tyrosine
Tyramine
N-Methyl-tyramine
Hordenine
Phenol
3-Amino-3-(4-hydroxyphenyl)-propanoate
4-Coumarate
4-Hydroxy-phenylpyruvate
(R)-3-(4-Hydroxy-phenyl)lactate
2-Hydroxy-3-(4-hydroxy-phenyl)propenoate
4-Hydroxy-phenylacetaldehyde
4-Hydroxy-phenylethanol
Salidroside
Phenylalanine metabolism
3-Hydroxy-phenylacetate
3,4-Dihydroxy-phenylacetate
Rosmarinate
Homogentisate
Gentisate
Gentisate-aldehyde
Hydroquinone
4-Hydroxy-phenylacetate
4-Hydroxy-phenylacetyl-CoA
4-Hydroxyphenyl-acetylglycine
4-Hydroxyphenyl-acetylglutamic acid
3-Maleyl-pyruvate
3-Fumaryl-pyruvate
4-Maleyl-acetoacetate
4-Fumaryl-acetoacetate
Fumarate
Pyruvate
Malate
Acetoacetate
Butanoate metabolism
Pyruvate metabolism
Citrate cycle
2-Hydroxy-5-carboxy-methylmuconate semialdehyde
5-Carboxymethyl-2-hydroxymuconate
5-Carboxy-2-oxohept-3-enedioate
2-Hydroxyhepta-2,4-dienedioate
2-Oxohept-3-enedioate
2,4-Dihydroxyhept-2-enedioate
Succinate semialdehyde
Succinate
HpaG
HpaI
4-Chloro-phenylacetate
Dioxin degradation
Dopaquinone
Leucodopaquinone
Dopachrome
Cysteinyl-DOPA
Pheomelanin
Melanin biosynthesis
Indole-5,6-quinone
Eumelanin
5,6-Dihydroxy-indole
DHICA
TYRP1
5,6-IQCA
4-(L-Alanin-3-yl)-2-hydroxy-cis,cis-muconate 6-semialdehyde
Stizolobate
5-(L-Alanin-3-yl)-2-hydroxy-cis,cis-muconate 6-semialdehyde
Stizolobinate
L-DOPA
3-(3,4-Dihydroxy-phenyl)pyruvate
3,4-Dihydroxy-phenylpropanoate
3-(3,4-Dihydroxy-phenyl)lactate
Dopamine
3-Methoxy-tyramine
Isoquinoline alkaloid biosynthesis
3,4-Dihydroxy-phenylacet-aldehyde
3-Methoxy-4-hydroxyphenyl-acetaldehyde
Homovanillate
L-Noradrenaline
L-Nor-metanephrine
L-Adrenaline
L-Metanephrine
3,4-Dihydroxy-mandelaldehyde
3-Methoxy-4-hydroxy-phenylglycolaldehyde
3,4-Dihydroxy-phenylethyl-eneglycol
3,4-Dihydroxy-mandelate
3-Methoxy-4-hydroxy-phenylethyleneglycol
3-Methoxy-4-hydroxymandelate
00350 9/1/21
(c) Kanehisa Laboratories

D

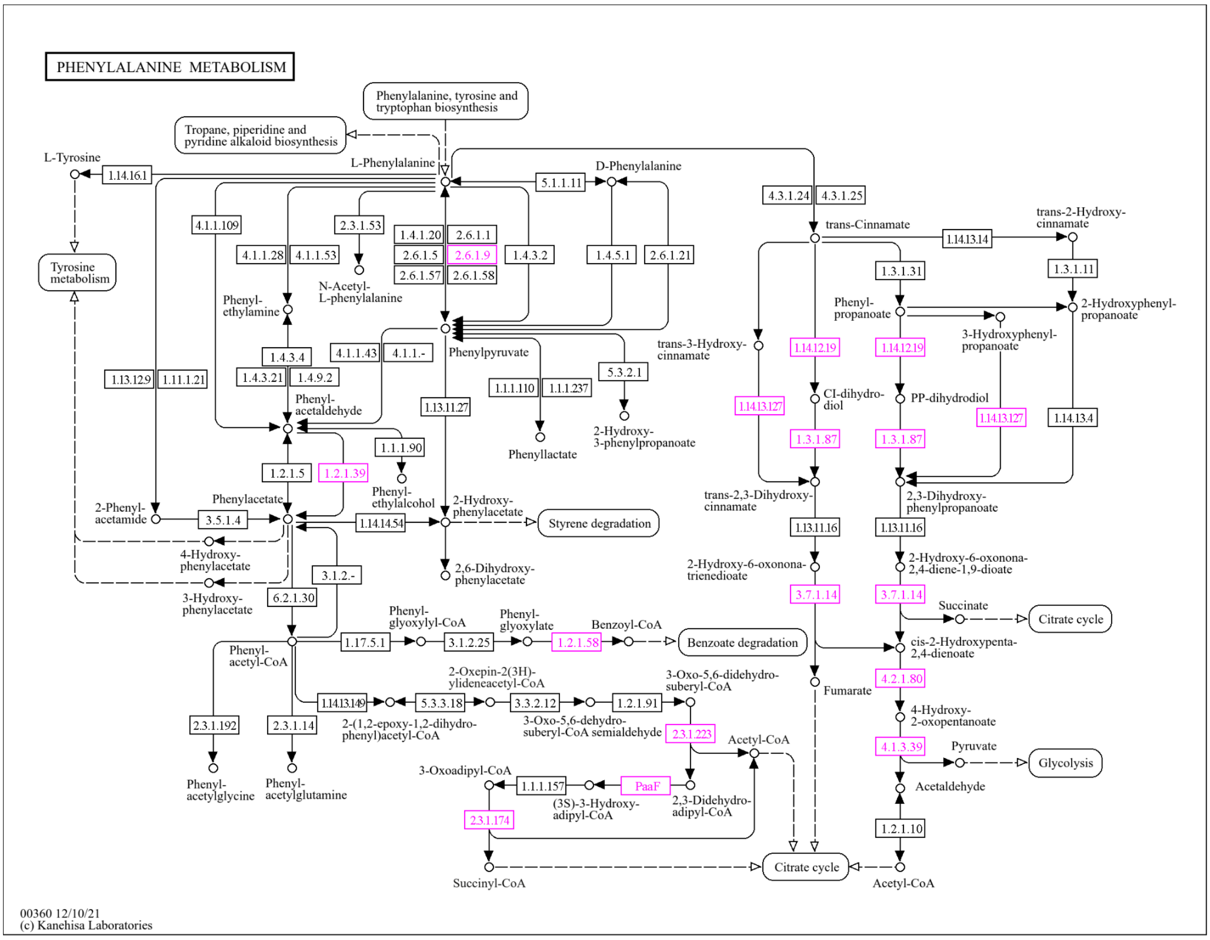

Figure S11: Degradation pathway maps. **A)** The following functional orthologs involved in toluene degradation were recovered: K03268, K18089, K07550, K16246, K00141, K03381, K18090. **B)** The following 9 functional orthologs involved in naphthalene degradation were recovered: K14582, K04072, K00480, K18242, K14581, K14580, K00152, K14583, K14584. **C)** The following functional 9 orthologs involved in Tyrosine metabolism [PATH:ko00350] were recovered: K00146, K00151, K02509, K16165, K00450, K04072, K00817, K01800, K01801. **D)** The following 12 functional orthologs involved in Phenylalanine metabolism [PATH:ko00360] were recovered: K02615, K00146, K05711, K05714, K05708, K00817, K02554, K05710, K01692, K01666, K00529, K18359, K05712.

Supplementary figure 12

A

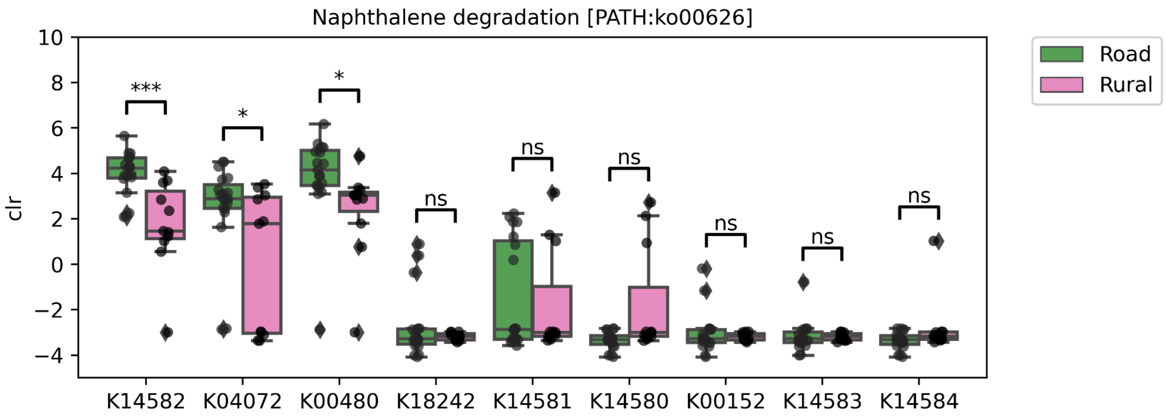

B

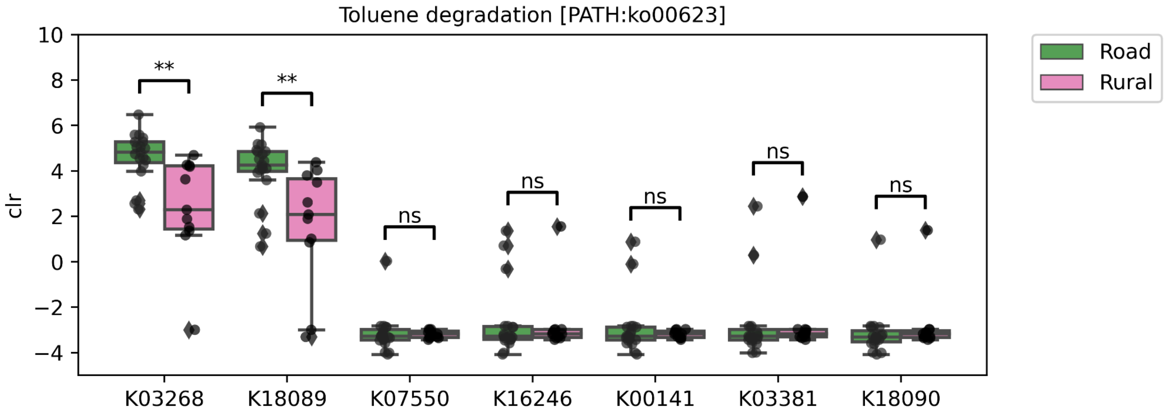

C

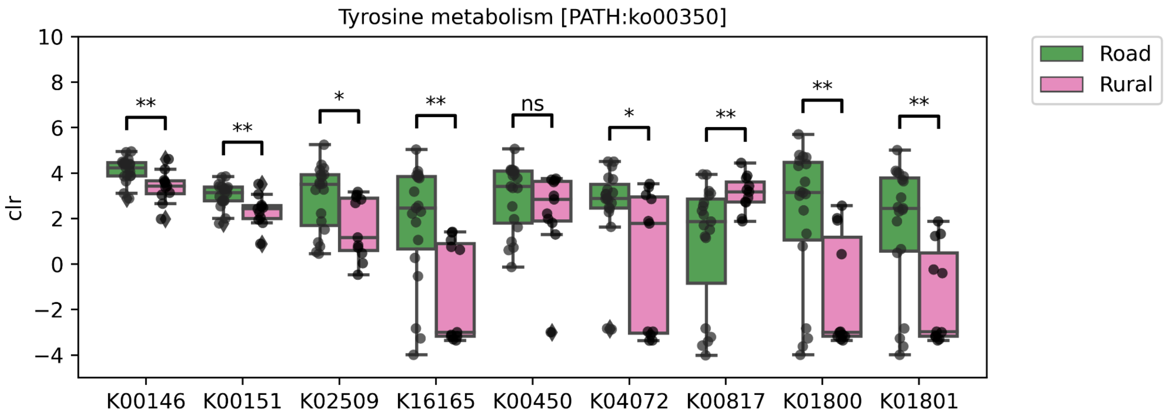

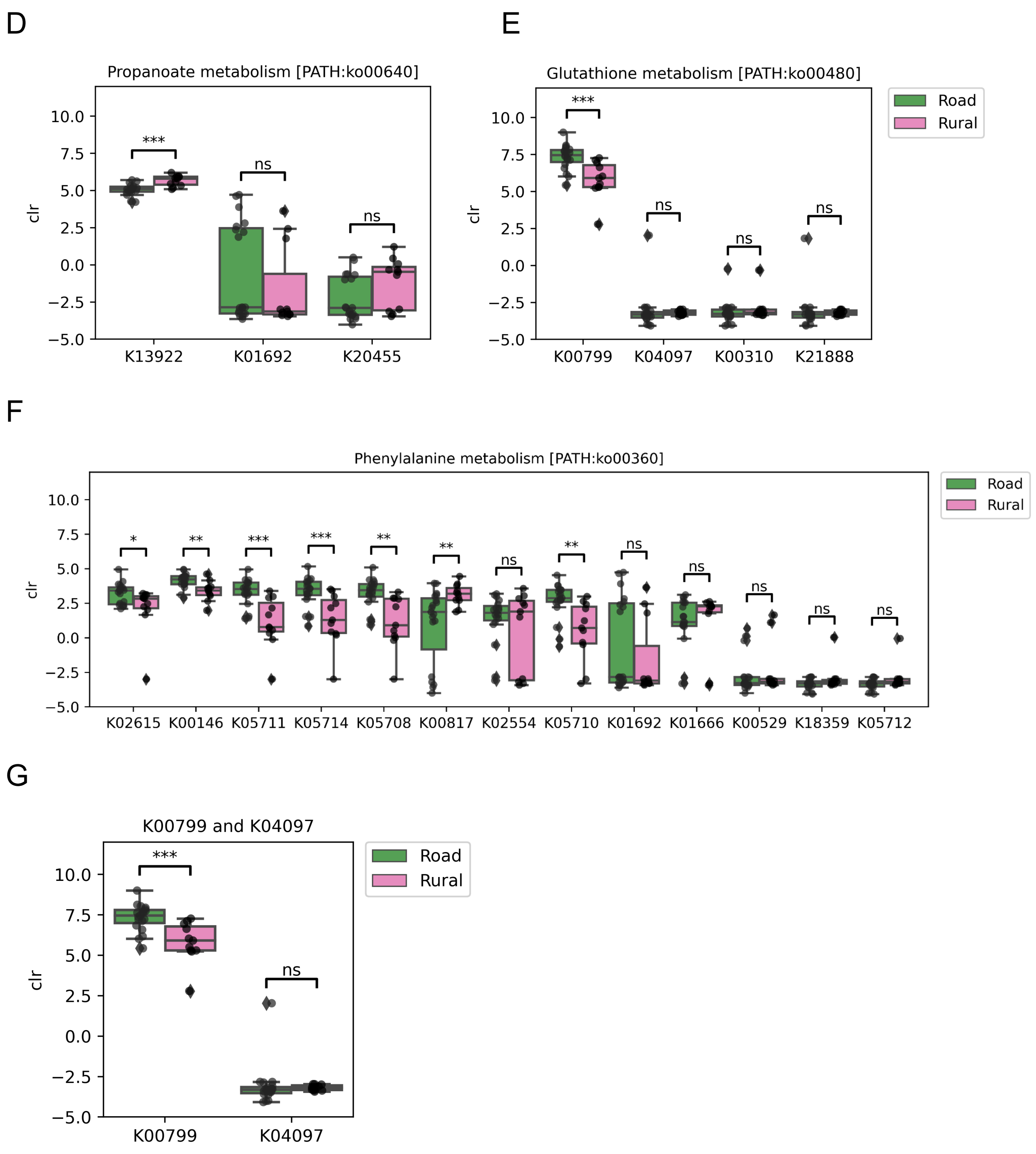

Figure S12: Difference in functional orthologs recovered from KEGG pathways. Significance tested with an independent t-test independent or a Welch t-test if the assumption of equal variances was not met $*P < 0.05$, $**P < 0.01$, $***P < 0.001$. **A)** Functional orthologs in naphthalene degradation. **B)** Functional orthologs toluene degradation. **C)** Functional orthologs in tyrosine metabolism. **D)** Functional orthologs in propanoate metabolism. **E)** Functional orthologs in glutathione metabolism. **F)** Functional orthologs in phenylalanine metabolism. **G)** Two functional orthologs recovered and part of a number of different pathways. K00799 was the only ortholog recovered from the following pathways: Drug metabolism - other enzymes [PATH:ko00983], Fluid shear stress and atherosclerosis [PATH:ko05418], Hepatocellular carcinoma [PATH:ko05225], Pathways in cancer [PATH:ko05200], Chemical carcinogenesis - receptor activation [PATH:ko05207], Platinum drug resistance [PATH:ko01524]. Both K007799 and K0497 were recovered from Chemical

carcinogenesis - DNA adducts [PATH:ko05204] and Drug metabolism - cytochrome P450 [PATH:ko00982].
